# Generation of a Transgenic Zebrafish Model for Pancreatic Beta Cell Regeneration

**DOI:** 10.31661/gmj.v8i0.1056

**Published:** 2019-11-06

**Authors:** Hossein Pourghadamyari, Mohammad Rezaei, Mohsen Basiri, Yaser Tahamtani, Behrouz Asgari, Seyedeh-Nafiseh Hassani, Reza Meshkani, Taghi Golmohammadi, Hossein Baharvand

**Affiliations:** ^1^Department of Biochemistry, Faculty of Medicine, Tehran University of Medical Sciences, Tehran, Iran; ^2^Fishery Faculty, Gorgan University of Agriculture Science and Natural Resources, Gorgan, Iran; ^3^Department of Stem Cells and Developmental Biology, Cell Science Research Center, Royan Institute for Stem Cell Biology and Technology, ACECR, Tehran, Iran; ^4^Department of Developmental Biology, University of Science and Culture, Tehran, Iran

**Keywords:** Diabetes, Pancreatic Beta Cells, Regeneration, Genetically Modified Animals

## Abstract

**Background:**

Diabetes is a major worldwide health problem. It is widely accepted that the beta cell mass decreases in type I diabetes (T1D). Accordingly, beta cell regeneration is a promising approach to increase the beta cell mass in T1D patients. However, the underlying mechanisms of beta cell regeneration have yet to be elucidated. One promising avenue is to create a relevant animal model to explore the underlying molecular and cellular mechanisms of beta cell regeneration. The zebrafish can be considered a model in beta cell regeneration studies because the pancreas structure and gene expression pattern are highly conserved between human and zebrafish.

**Materials and Methods:**

In this study, the Tol2 transposase was exploited to generate a Tg(*Ins:egfp-nfsB*) zebrafish model that expressed a fusion protein composed of enhanced green fluorescent protein (EGFP) and nitroreductase (NTR) under control of the *Ins* promoter.

**Results:**

Metronidazole (MTZ) treatment of Tg(*ins:egfp-nfsB*) zebrafish larvae led to selective ablation of beta cells. Proof-of-concept evidence for beta cell regeneration in the transgenic larvae was observed two days after withdrawal of MTZ.

**Conclusion:**

This study suggests that the Tg(*ins:egfp-nfsB*) zebrafish can be used as a disease model to study beta cell regeneration and elucidate underlying mechanisms during the regeneration process.

## Introduction

Type I diabetes (T1D) is a chronic autoimmune disease in which cell-mediated autoimmune reactions destroy the insulin-producing cells (beta cells). Although the primary causes of T1D development are unknown, it is widely accepted that approximately 70%–80% of the beta cell mass is lost or damaged in T1D patients. Patients with T1D comprise approximately 10% of the diabetic population worldwide [1]. Currently, several approaches are available to compensate for destroyed beta cells in patients with T1D. One of the newly recommended treatments for T1D is to compensate for the loss of beta cells [2, 3]. Current approaches include pancreas transplantation [4], pancreatic islet transplantation [5, 6], stem cell differentiation [7], and beta cell regeneration [3]. However, organ donor shortage and adverse effects of corticosteroids are two main problems that decrease the feasibility of transplantation approaches [1]. Moreover, up to now, there is no strong, comprehensive protocol for successful stem cell differentiation toward mature pancreatic beta cells [8]. Beta cell regeneration is one of the most important approaches used for this purpose. Beta cell regeneration is an attractive, hopeful approach that can be used to increase the beta cell mass in type 1 diabetic patients. However, the underlying mechanisms of beta cell regeneration remain to be elucidated [9, 10]. One promising avenue is to create a relevant animal model to explore the underlying molecular and cellular mechanisms of beta cell regeneration. The advancements in transgenic animal models pave the way for modeling human diseases [11, 12]. Pancreatic structure and gene expression patterns are highly conserved between zebrafish and human [13–17]. The zebrafish (*Daniorerio*) is a small tropical freshwater fish with special features that make it suitable for genetic and regeneration studies. These features transparency at the early developmental stages, easy husbandry and care, and regenerative capacity for most organs, along with extremely fast development. Recent advances in genetic engineering provide various strategies to produce transgenic zebrafish models. The Tol2 transposase method is one of the most popular approaches for the production of transgenic animals [15, 18]. Tol2 transposase was introduced by Koga *et al*. in 1996. They have shown that, unlike other transposons, Tol2 elements are autonomously active [19]. Several studies reported the benefit of the Tol2 transposase system for a wide range of investigations in the field of genome manipulations, such as gene transfer and transgenic technology [20–22]. On the other hand, nitroreductase (NTR) enzymes encoded by the *nfsB* gene in *Escherichia coli* (*E. coli* str. K-12) can metabolize polynitroaromatic compounds. Previous studies confirmed that metronidazole (MTZ), as a popular NTR substrate, could be used for ablation of cells in animal models. MTZ is metabolized and converted to toxic substances by the NTR enzyme, which thereby can induce apoptosis [23–25]. We designed the current study to produce Tg(*ins:egfp-nfsB*) transgenic zebrafish as an animal model for use in beta cell regeneration studies.

## Materials and Methods

### Zebrafish Maintenance, Embryo Collection, and Microinjection

We used TU strain zebrafish in this study. The animals were maintained according to standard conditions in a circulating system that filtered wastewater and maintained fresh, clean environmental water. Zebrafish were kept in an aquarium at 28.5°C under a 10/14-h dark/light cycle. Embryos were collected, kept at 28.5°C, and staged according to days post-fertilization (dpf). Zebrafish were fed a commercial diet twice a day. To obtain zebrafish zygotes, we placed the selected female and male zebrafish into breeding tanks separated by a divider. The ratio of female to male zebrafish in each breeding tank was 2:1. The tanks were left in the dark at 28.5°C overnight. On the next morning, we removed the divider, and 10 minutes after spawning, the embryos were collected and washed. Good-quality embryos were selected for genetic construct microinjections. The institutional animal care and use committee of Royan Institute approved the animal protocols.

### Genomic Polymerase Chain Reaction (PCR)

Genomic DNA was extracted from larvae samples using a DNA extraction Kit in accordance with the provided instructions­­ (Qiagen, Germany). PCR amplification was performed by Platinum® Taq DNA polymerase high fidelity (Invitrogen, USA) using a thermal cycler (Eppendorf, Germany) in a final volume of 12.2 µl under standard conditions. We added 38 µl of autoclaved, distilled water to the contents of each microtube. Each microtube contained 0.2 µM each of one of the primers, 0.8 mM of dNTPs (Invitrogen, USA), 2 mM of MgCl_2_, and 0.2 µl of Platinum® Taq DNA Polymerase High Fidelity (Invitrogen, USA). Finally, 200 ng of genomic DNA was added. The cycling conditions were: 94°C for 4 min followed by 35 cycles that comprised 94°C for 30 sec, an annealing step at 58°C for 45 sec, and extension at 68°C for 30 sec with a final extension at 68°C for 10 min.

### Genetic Constructs

First, PCR cloning was performed to insert the zebrafish insulin promoter (gene ID: 30262) into the Royan Tol2 enhanced green fluorescent protein (EGFP) 2A plasmid. Accordingly, forward and reverse primers with SphI and SalI restriction sites were designed to amplify 1000 bp upstream of the insulin coding sequence (CDS). The purified PCR product was ligated by using the Gel Extraction Kit (Macherey-Nagel) into the Tol2 EGFP cloning vector before the EGFP sequence. A second PCR cloning was applied in order to insert the *nfsB* CDS (gene ID: 945778) into the Tol2 recombinant plasmid. For this purpose, we designed *nfsB* primers, and NdeI and EcoRI restriction sites were flanked to 5ʹ of the forward and reverse primers, respectively. Also, the purified PCR product was cloned into the Tol2 recombinant plasmid after the EGFP sequence. Both cloning PCR results were confirmed by digestion and sequencing, respectively. The NCBI Primer-BLAST (https://www.ncbi.nlm.nih.gov/tools/primer-blast/) was used as the primer designing tool (Table-1). We prepared transposase mRNA by the in vitro transcription method. To this end, a pCS2-transposase plasmid (gifted by Dr. Ekker, Ottawa, Canada) was linearized and precipitated. Next, the in vitro transcription process was conducted by using the mMESSAGE mMACHINE SP6 Transcription Kit (Life Technologies, USA). The product of this process was treated by DNase I to eliminate the plasmid DNA. Finally, lithium chloride (LiCl) precipitation was performed to remove unincorporated nucleotides and proteins. The quality of the mRNA product was analyzed by agarose gel electrophoresis. Tol2 recombinant plasmid and transposase mRNA were co-injected into the one-cell stage of the zebrafish zygotes at concentrations of 50 ng/ul and 100 ng/ul. The injected embryos were evaluated at 3dpf by using a fluorescence stereo-microscope in order to select the embryos that expressed EGFP in their pancreases. EGFP positive embryos were then chosen and raised as the F0 generation of Tg*(ins:egfp-nsfB*) zebrafish. Then, each one of the F0 generation adult zebrafish were crossed with a wild-type (TU stain) zebrafish to produce the F1 generation.

### Reverse Transcription QuantitativePCR (RT-PCR)

Total RNA was extracted using TRIzol (Sigma, USA). DNase (Fermentas, Germany) treatment was performed according to the manufacturer’s instructions. The amount and quality of RNA were assessed by spectrophotometry at 260 nm and agarose gel electrophoresis, respectively. Total RNA (1 µg) was reverse transcribed using reverse transcriptase enzyme (Takara) and random hexamer primers. Real-time PCR was performed on a Rotor-Gene 3000 Instrument (Corbett Research, Australia). Insulin and glucagon expression levels were measured by specific primers. The data were normalized against the *eef1a1l1* transcript level and analyzed by the delta-delta Ct method.

### MTZ Treatment

The beta cells of our Tg(*ins:egfp-nfsB*) zebrafish were ablated using MTZ as following: At 3dpf, the Tg(*ins:egfp-nfsB*) larvae were treated by 10 mM MTZ solution (Sigma) for 24 h at 28.5°C in the dark. After 24 h, we washed the larvae three times with E3 medium. Then, 5 ml of fresh E3 medium were added to each well of larvae. Beta cell regeneration analysis was carried out at the end of 6 dpf.

### Image Analysis

Fluorescent images were captured in 3dpf (before MTZ treatment), 4dpf (after MTZ treatment), and 6dpf (2 days after MTZ was washed out) using an Olympus microscope with a GFP filter (Olympus, Japan). All images were acquired under the same settings in terms of laser power and magnification. Finally, the GFP area and fluorescence intensity were quantified using ImageJ software (version 1.48, National Institutes of Health, USA).

### Statistical Analysis

The data are presented as mean±SD of at least three independent experiments. Statistical analyses were carried out using SPSS 13 (SPSS, USA). Comparisons among all groups were performed with one-way analysis of variance (ANOVA). If significant differences were found, a posthoc Bonferroni test was carried out.

## Results

**Figure 1:**
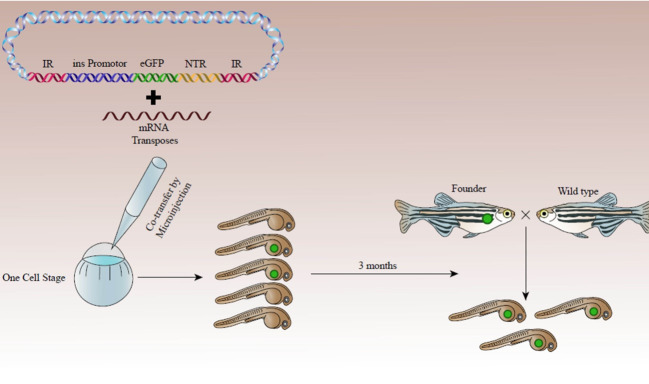
Schematic procedure for the generation of the transgenic zebrafish model for pancreatic beta cell regeneration. The recombinant Tol2 plasmid and transposase mRNA were co-injected into the one-cell stage of zebrafish zygotes. The injected embryos were evaluated at 2 dpf using fluorescence stereo-microscope, and EGFP positive embryos were selected. EGFP positive embryos raised as F0 generation of Tg(ins:egfp-nsfB) zebrafish. Then each one of the F0 generation adult zebrafish was crossed with a wild-type (TU stain) zebrafish to produce F1 generation.

A schematic procedure for generation of the transgenic zebrafish model for pancreatic beta cell regeneration is presented in Figure-1.

### Evaluation of Genetic Constructs and Transposase mRNA

**Figure 2:**
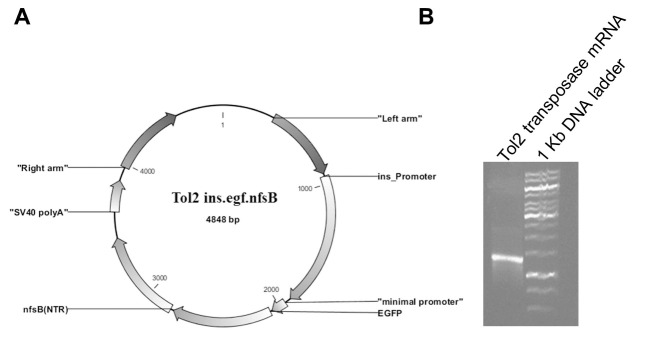
Transposon DNA construct and Tol2 transposase mRNA for generation of Tg(ins:egfp-ntr) zebrafish. **A:** Plasmid map of Tol2 recombinant plasmid. **B:** In vitro transcription of transposase mRNA after DNase treatment (DNA size marker is Fermentas 1 kb DNA ladder).

We performed two PCR clonings with a standard molecular biology protocol to insert the insulin promoter and *nfsB* sequence into the Tol2 plasmid (Figure-2A). Digestion and sequencing analysis results confirmed insertion of the insulin promoter and *nfsB* sequence into the Tol2 plasmid. Gel agarose electrophoresis confirmed the appropriate quality of the transposase mRNA (Figure-2B).

### Production and Evaluation of FunderTg(ins:egfp-nfsB) Zebrafish

Tol2 recombinant plasmid and transposase mRNA were co-injected in 272 one-cell-stage embryos. Fluorescence stereo-microscope analysis indicated that 14 embryos expressed EGFP on their pancreases. The EGFP positive embryos were raised and crossed with wild-type fish to survey the rate of germline transmission. Of 14 EGFP positive embryos, only one showed germinal transmission.

### Tissue-Specific NTR/MTZ Cell Ablation

**Figure 3:**
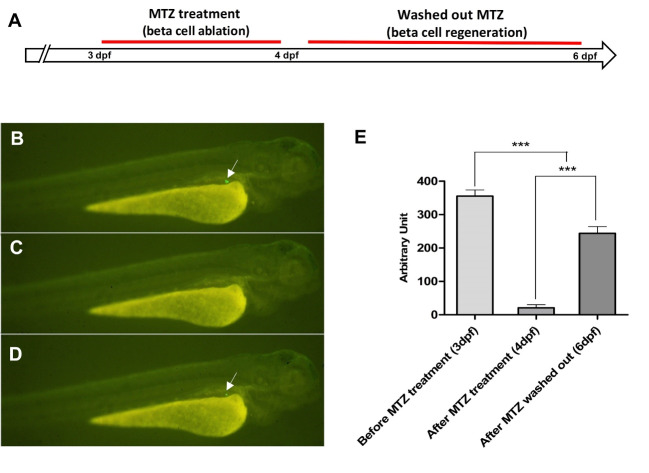
MTZ treatment specifically depletes beta cells of and beta cell regeneration. **A:** Schematic diagram for cell-labeling and assessment of beta cell regeneration. **B:** Before MTZ treatment (3dpf). **C:** After 24 hrs MTZ treatment (4dpf) ablate the beta cells. **D:** Expression of EGFP appeared again after 2-day MTZ withdrawal (6dpf). Arrows show the beta cells. **E:** Quantitative analysis GFP intensities at 3 dpf, 4 dpf, and 6 dpf (n=5). ***P<0.001.

Beta cells were targeted for ablation from 3 to 4 dpf by using the beta cell-specific expression of NTR (Figure-3A). NTR converts MTZ into a cytotoxic product. After 24 h of MTZ treatment (4dpf), we observed beta cell ablation by decreased EGFP expression (Figure-3B and C). We observed that *nfsB* encoded NTR induced drug dependent apoptosis in these cells.

### Beta Cell Regeneration Following Ablation

We sought to determine whether the Tg(*ins:egfp-nfsB*) transgenic fish could be used as a model of beta cell regeneration. Hence, we evaluated GFP signals of the MTZ treatment larvae after 2 days of recovery by fluorescent stereomicroscope photos. Data showed the increment in GFP signals after 2 days of recovery (Figure-3D). The fluorescent images at 4 dpf (after MTZ treatment) and 6 dpf (2 days after MTZ washed out) were analyzed and quantified by ImageJ software. The results of fluorescent images also revealed significant increases in EGFP intensity after 2 recovery days (6 dpf) compared to MTZ treatment larvae (Figure-3E).

### Evaluation of ins and gcga Expressions in Tg(ins:egfp-nfsB) Zebrafish

**Figure 4:**
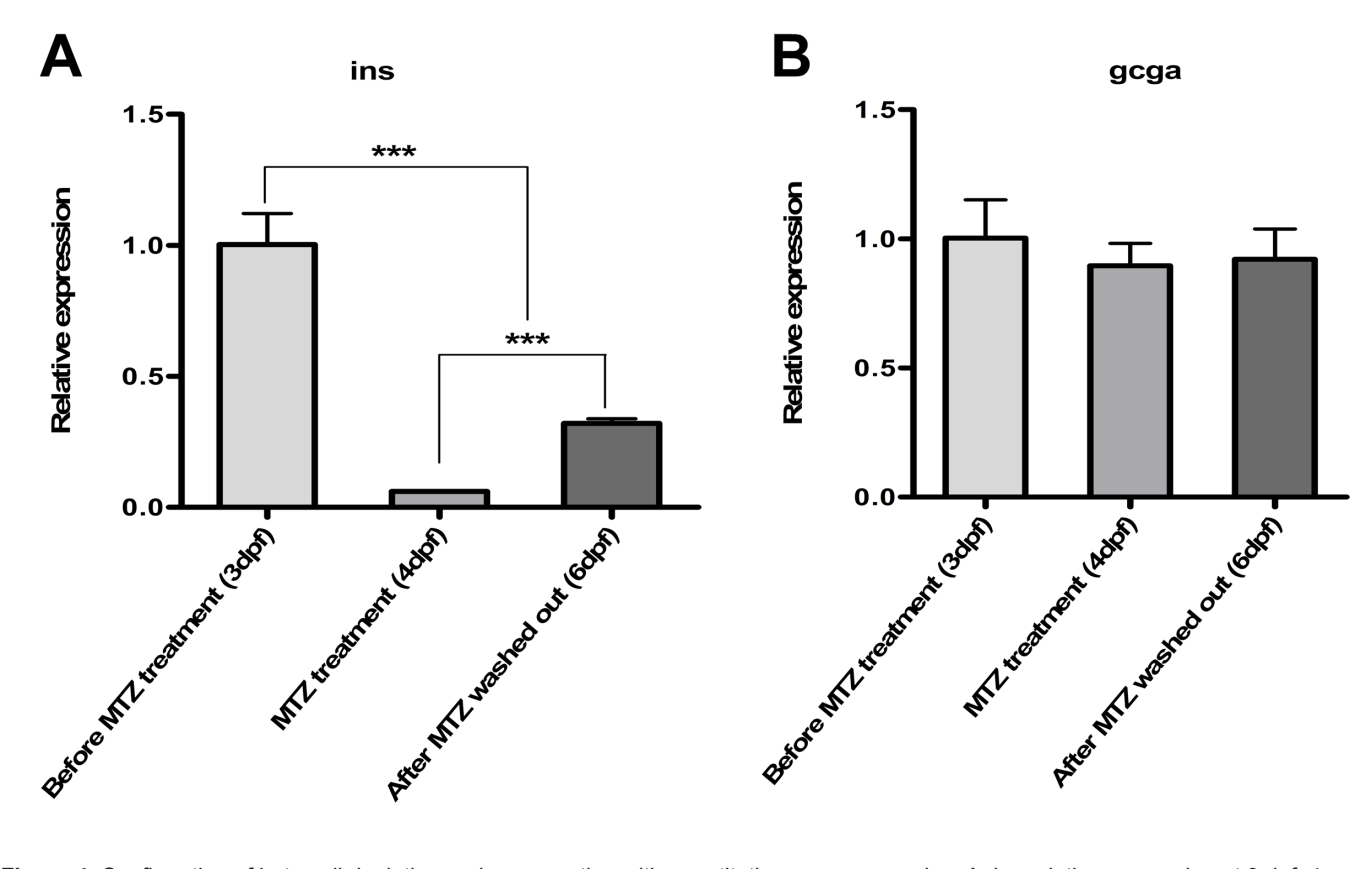
Confirmation of beta cell depletion and regeneration with quantitative gene expression. A: ins relative expression at 3 dpf, 4 dpf, and 6 dpf. B: gcga relative expression at 3 dpf, 4 dpf, and 6 dpf. mRNA levels were quantified normalized relative to eef1a1l1 mRNA expression. ***P<0.001.

In order to assess the function of the NTR/MTZ system in the Tg(*ins:egfp-nfsB*) zebrafish model, RNA was extracted before and post-treatment with MTZ. We performed expression analysis of *ins* and *gcga* by using RT-PCR. The result showed a significant decrease in *ins* expression in MTZ-treated embryos compared to non-MTZ-treated embryos. We observed no change in *gcga* expression (Figure-4A and B).

## Discussion

In this study, we used the Tol2 transposon method to produce Tg(*ins:egfp-nfsB*) zebrafish as a beta cell regeneration animal model. The efficiency of this study was low. Numerous studies reported that the efficiency of the Tol2 transposase was considerably more than 50% [26, 27]. According to a study by Rembold *et al*. in 2006, and Grabher and Wittbrodt in 2007, it seemed that the inconsistency in Tol2 transposase efficiency could be attributed to the differences in injection sites. Here, we injected the constructs into the yolks of zygotes. They reported that injection gene constructs in the yolk could have a less efficient Tol2 system [28, 29]. We added MTZ to the aquatic environment of the transgenic zebrafish to test the proper expression and efficiency of *nfsB* in beta cell ablation. Because both EGFP and *nfsB* expression is driven by the zebrafish insulin promoter, we have used fluorescent microscopic analysis to survey the ability of NTR on beta cell ablation. Fluorescent microscopic data revealed the proper function of the NTR/MTZ system in the Tg(*ins:egfp-nfsB*) zebrafish. Image analysis showed decreased EGFP intensity in MTZ-treated embryos compared to non-MTZ-treated embryos. These data suggested that NTR could induce beta cell ablation in the Tg(*ins:egfp-nfsB*) zebrafish model. Also, the increased EGFP intensity after washing out MTZ confirmed the potentiation of beta cell regeneration in the Tg(*ins:egfp-nfsB*) zebrafish model. These data were consistent with a study by Fang *et al*. in 2015 have shown that the NTR/MTZ system was effective for demyelination and re-myelination in the Tg(*mbp:nfsB-egfp*) zebrafish model [15]. Gene expression data analysis indicated a significant decrease in expression of *ins* in MTZ-treated embryos compared to non-MTZ-treated embryos. However, these data showed no any significant difference in expression of *gcga* between MTZ-treated and non-MTZ-treated embryos. It could be concluded from this data that MTZ treatment specifically destroyed only beta cells and did not have any bystander effect. We have observed that in Tg(*ins:egfp-nfsB*) zebrafish EGFP expressed under control of the zebrafish insulin promoter. Hence, the insulin-expressing cells were visualized. Therefore, beta cell ablation and regeneration of Tg(*ins:egfp-nfsB*) zebrafish were indirectly analyzed in this study [30].

## Conclusion

Altogether, these results suggested that the EGFP-NTR fusion protein specifically expressed in beta cells of the Tg(*ins:egfp-nfsB*) zebrafish model. The data indicated that the NTR/MTZ system has a specific function in beta cell ablation in the Tg(*ins:egfp-nfsB*) zebrafish model. Further immunohistological investigations using zebrafish insulin-specific antibody may provide more direct information about the details of beta cell ablation and regeneration in this transgenic model. Our data confirmed the ability of beta cell regeneration in this model. This Tg(*ins:egfp-nfsB*) zebrafish model could be used as a beta cell regeneration animal model.

## Acknowledgment

This work was supported by Royan Institute, the Iran National Science Foundation (INSF), Iran Science Elites Federation to H. B (Grant number : 94000076) ,and Tehran University of Medical Sciences(Grant number: 28514).

## Conflict of Interest

No conflict of interest.

**Table 1:** The Sequences of Primer Used

**Primer name**	Primer sequences*
*ins*	F: AGAGGC**ATGCTG**TTTGTACCATAATAAGCT
	R: AGAG**GTCGAC**TCACACTGACACAAACACAC
*nfsB*	F: TAGC**GTCGAC**ATGGATATCATTTCTGT
	R: TAGC**GGATCC**CACTTCGGTTAAGGTGATGT
*ins*	F: AGAGAGACGTTGAGCCCCTT
	R: GCACTGCTCTACAATGCCTC
*gcga*	F: GCGTCCAGTATTTTGCCAGTC
	R: CATGGTCGTCAAACCCGGAG
*eef1a1l1*	F: TACCCTCCTCTTGGTCGCTT
	R: GAAGAACACGCCGCAACCTT

* Restriction sites are underlined.
